# Aberrant DNA Polymerase Beta Enhances *H. pylori* Infection Induced Genomic Instability and Gastric Carcinogenesis in Mice

**DOI:** 10.3390/cancers11060843

**Published:** 2019-06-18

**Authors:** Shengyuan Zhao, Megha Thakur, Alex W. Klattenhoff, Dawit Kidane

**Affiliations:** Division of Pharmacology and Toxicology, College of Pharmacy, Dell Pediatric Research Institute, The University of Texas at Austin, 1400 Barbara Jordan Blvd. R1800, Austin, TX 78723, USA; zhaosy@utexas.edu (S.Z.); meghathakur@utexas.edu (M.T.); alexklattenhoff@utexas.edu (A.W.K.)

**Keywords:** mutation in DNA polymerase beta, genomic instability, *H. pylori*, gastric cancer

## Abstract

*H. pylori* is a significant risk factor of gastric cancer that induces chronic inflammation and oxidative DNA damage to promote gastric carcinoma. Base excision repair (BER) is required to maintain the genome integrity and prevent oxidative DNA damage. Mutation in DNA polymerase beta (Pol β) impacts BER efficiency and has been reported in approximately 30–40% of gastric carcinoma tumors. In this study, we examined whether reduced BER capacity associated with mutation in the *POLB* gene, along with increased DNA damage generated by *H. pylori* infection, accelerates gastric cancer development. By infecting a Pol β mutant mouse model that lacks dRP lyase with *H. pylori*, we show that reactive oxygen and nitrogen species (RONS) mediated DNA damage is accumulated in Pol β mutant mice (L22P). In addition, *H. pylori* infection in Leu22Pro (L22P) mice significantly increases inducible nitric oxide synthesis (iNOS) mediated chronic inflammation. Our data show that L22P mice exhibited accelerated *H. pylori* induced carcinogenesis and increased tumor incidence. This work shows that Pol β mediated DNA repair under chronic inflammation conditions is an important suppressor of *H. pylori* induced stomach carcinogenesis.

## 1. Introduction 

*H. pylori* infection is considered the main risk factor for gastric cancer [1,2,3]. In this case, the predisposing inflammation is most often caused by colonization of the gastric epithelium by *Helicobacter pylori (H. pylori)* and chronically infected individuals have an increased risk of developing gastric cancer [3,4]. *H. pylori* associated chronic inflammation induces immune and epithelial cells to release reactive oxygen and nitrogen species (RONS), which are capable of causing DNA damage [5,6]. *H. pylori* infection also promotes oncogene activation [7,8] and cellular proliferation [9,10]. Furthermore, *H. pylori* induced chronic inflammation exhibits an increase infiltration of macrophages and neutrophils that leads to increased levels of RONS [11]. RONS in turn induce base lesions including 8-oxoGuanine (8-oxoG), which have been observed at sites of inflammation [11,12]. In addition, *H. pylori* infection inhibits DNA repair proteins, including mismatch repair proteins and base excision repair (BER) proteins, which play a major role in maintaining the genome integrity [13,14,15]. 

BER is a major DNA repair pathway that removes the majority of oxidative DNA damage without affecting the double helix DNA structure [16,17,18]. Oxidative DNA damage repair via BER is the primary repair pathway that protects against oxidative DNA damage [16]. BER is initiated by recognition and excision of the damaged base by specific DNA glycosylases including OGG1, endonuclease III–like protein 1 (NTH1) and Nei-like proteins (NEIL1, NEIL2 and NEIL3) (7, 8). DNA glycosylases recognize and remove specific types of DNA base damage, leaving abasic sites (AP sites). The essential enzyme apurinic/apyridimic endonuclease (APE1) recognizes the AP sites and cleaves the DNA backbone at the 5′ side of the lesion to generate a 3′ hydroxyl and a 5′ deoxyribose phosphate (5′d-RP) flap. Subsequently, the DNA gaps are filled by DNA Pol β and nick sealed by a DNA ligase III and XRCC1 or via ligase I [19]. 

Previous studies have shown that various genetic alterations occur in the gastro-mucosa during chronic gastritis [20,21], suggesting that the accumulation of genetic mutations induced by *H. pylori* infection leads to development of gastric cancer. Host BER capacity could modify the process of carcinogenesis of *H. pylori*. Few studies have shown that aberrant BER contributes to infection induced genomic instability and tumorigenesis. However, there is little experimental evidence that provides mechanistic insight into how aberrant host BER contributes to *H. pylori* induced genomic instability and carcinogenesis. Furthermore, the impact of aberrant Pol β in BER function during *H. pylori* infection has not been shown. To further investigate the role of BER in protecting the genome from *H. pylori* induced oxidative damage, we used *H. pylori* infection and a Pol β mutant mouse model that lacks dRP lyase function to determine whether Pol β mediated BER helps to maintain genomic integrity and prevent *H. pylori* induced carcinogenesis. For this purpose, we used a Pol β mutant mouse model and *H. pylori* cagA positive strains. Our data show that upon *H. pylori* infection, Pol β mutant mice exhibit increased accumulation of oxidative DNA damage that likely exacerbates genomic instability and ultimately leads to decreased tumor latency. Overall, our data provide mechanistic insight into how infection related aberrant BER contributes to genomic instability and carcinogenesis. 

## 2. Results 

### 2.1. POLB Mutation Does Not Affect H. pylori Colonization in Mice Stomach 

**Figure 1:**
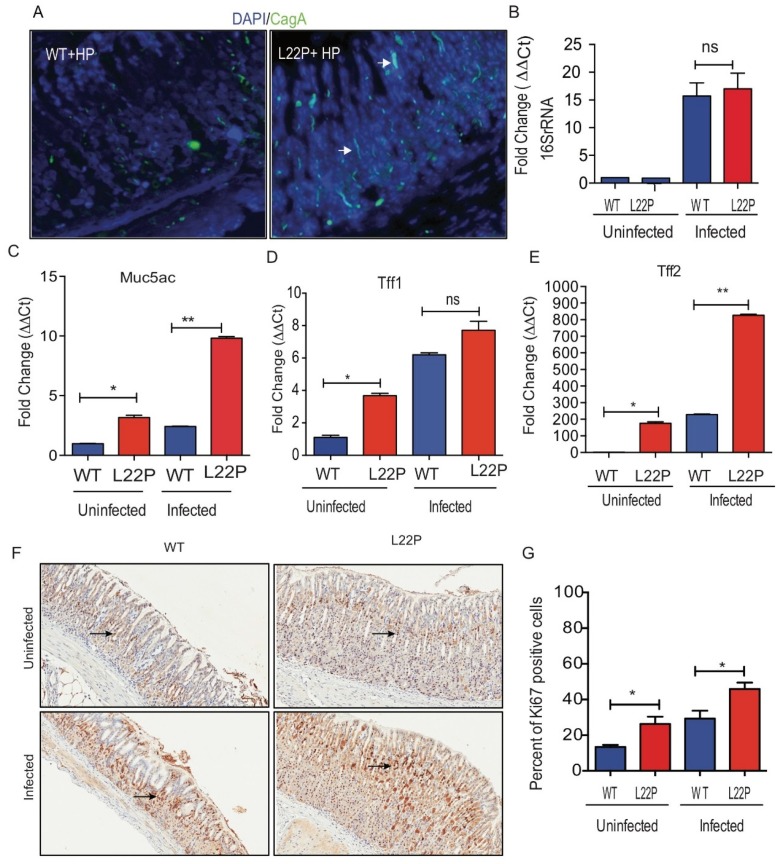
Mutation in *POLB* gene do not alter *H. pylori* colonization in stomach. (**A**) Immunocytochemistry of *CagA* localization of H. pylori on stomach tissues section from WT and L22P after 7 days of infection; (**B**) The quantitative reverse transcription polymerase chain reaction (qRT-PCR) of 16S rRNA to measure the copy number of *H. pylori* 16S rRNA in WT and L22P infected versus uninfected mice; (**C**) Gene expression of MUC5AC from surface epithelium of stomach tissues; (**D**) Tff1 mRNA expression; (**E**) Tff2 mRNA expression; (**F**,**G**) Immunohistochemistry staining of with proliferation marker ki67; (**G**) Estimated percent of cells positive for ki67staining. All data were analyzed using GraphPad prism (GraphPad Prism Inc, San Diego CA, USA). * *p* < 0.05; ** *p* < 0.01 and “ns” represent non-significant difference.

We investigated whether the stomach microenvironment of Leu22Pro (L22P) mice favors the *H. pylori* colonization better than wild-type (WT) mice. We stained stomach tissues sections, from L22P and WT mice infected with *H. pylori*, with *CagA* (cytotoxin associated pathogen) antibody and there was no difference in the number of *CagA* positive *H. pylori* between L22P versus WT mice stomach (Figure 1A). In addition, we observed no significance difference in copy number of 16SrRNA of *H. pylori* between L22P and WT mice stomach (Figure 1B). In contrast, we found that the secreted mucin MUC5AC, which is a major component of the mucinous layer lining the gastric epithelium significantly increased in *H. pylori* infected L22P mice versus *H. pylori* infected WT mice (Figure 1C). Furthermore, TFF2 expression significantly increased in L22P mice versus WT (Figure 1D,E, *p* < 0.001), suggesting that gastric mucous cells likely display precancerous stage of tumor initiation. To determine whether L22P mutation increases cell proliferation in gastric cells, we measured the level of ki-67 positive cells with immunohistochemistry staining and we found that the percent of ki-67 positive cells significantly increased in L22P versus WT mice infected with *H. pylori* (Figure 1F,G; *p* < 0.05). Furthermore, the association between *H. pylori* and cancer may be attributable to increased epithelial cell turnover. We found moderate increase in apoptosis in L22P versus WT mice stomach (Supplementary Materials Figure S1).

### 2.2. POLB Mutation Exacerbates H. pylori Infection Induced Oxidative DNA Damage in Mice Stomach

**Figure 2:**
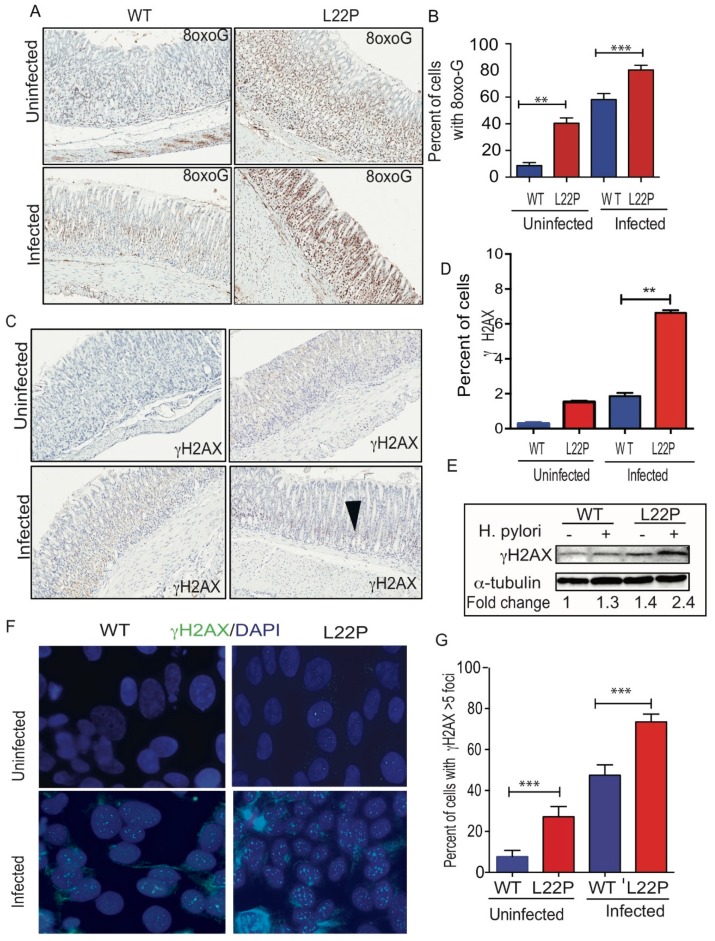
Mutation in *POLB* exacerbates *H. pylori* induced genomic instability in mice. (**A**) Representative staining of 8oxoG on WT and L22P cells with and without *H. pylori* infection; (**B**) Estimated percent of cells positive for 8oxoG lesions on WT and L22P with and without infection; (**C**) Immunohistochemistry staining of γH2Ax in WT and L22P infected with *H. pylori*; (**D**) Estimated percent of positive cells for γH2AX in WT and L22P infected and non-infected groups; (**E**) Western blot for γH2AX on WT and L22P mice, with and without infection; (**F**) Localization of γH2AX in normal human gastric epithelial cells (GES-1) that expressed WT and L22P infected with *H. pylori*; (**G**) Estimated percent of cells with γH2AX positive with and without infected WT and L22P expressing human gastric epithelial cells (GES-1); (**H**) Cell cycle dependent accumulation of double strand breaks in *H. pylori* infected GES-1cells. Cells were co-cultured and stained with γH2AX antibody and the cell cycle analysis was performed using flow cytometry; Data analysis was done using Two way ANOVA; (**I**) Representative image of transformed cells after *H. pylori* infection for 15 days co-culture; and estimated number of foci/field for transformed cells that were infected with *H. pylori* for 15 days. All statistical analysis was performed using the paired *t*-test on GraphPad prism software. The statistical significances represented as ** *p* < 0.01 and *** *p* < 0.001.

To assess a possible effect of *H. pylori* infection on DNA integrity of gastric cells, we measured the level of 8-oxoG and **γ**H2AX in stomach tissue (Figure 2). We found that the percent of gastric cells positive for 8oxoG lesions significantly increased in L22P versus WT mice infected with *H. pylori* (Figure 2A,B, WT (18.91%); L22P (59.11%); *p* < 0.0001). Further, we examined whether *H. pylori* infection exacerbate double strand breaks (DSBs), we found that the number of positive cells for **γ**H2AX foci was significantly increased in L22P mice versus WT infected with *H. pylori* (Figure 2C,D, WT (1.53%) versus L22P (6.63%); *p* < 0.0001). In addition, protein extract from stomach tissue shows that the level of **γ**H2AX expression increases in L22P infected mice (Figure 2E). Furthermore, our in-vitro experiment showed that normal human gastric epithelial cells (GES-1) expressing mutant *POLB* proteins infected with *H. pylori* infection exhibits a significant increase in DSBs (Figure 2F,G, *p* < 0.001). Next, we quantified the accumulation of DSBs after infection at different stage of cell cycle. We found that cells with DSBs significantly accumulated at S and G2 stage of cell cycle after *H. pylori* infection in L22P versus infected WT cells (Figure 2H, *p* < 0.0001). This result supports the notion that *H. pylori* infection may generate other BER intermediates (AP sites) to block replication fork that lead to accumulation of DSBs in S/G2 stage of L22P cells. Furthermore, to determine genomic instability induces cellular transformation in-vitro, we performed co-culture experiment as and found that foci formation significantly increases in L22P infected cells versus WT infected cells (Figure 2I, *p* < 0.01).

### 2.3. Mutation in POLB Exacerbates Inflammatory Response in Stomach

**Figure 3:**
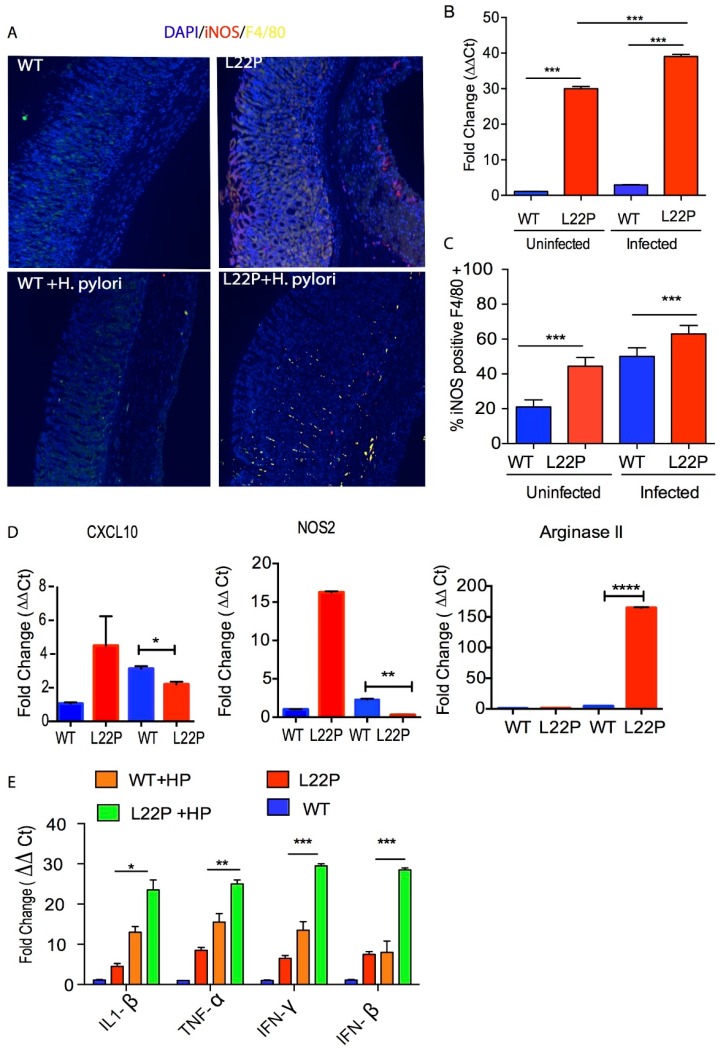
*H. pylori* infection synergize with *POLB* mutation to increase inflammatory response. (**A**) Localization of iNOS producing macrophage using immunocytochemistry, macrophage stained with F4/80 antibody (green stain) and iNOS stained with antibody (red stain); (**B**) Gene expression of iNOS using qRT-PCR from WT and L22P mice stomach tissue extract with and without *H. pylori* infections; (**C**) Estimated percent of iNOS producing macrophage (F4/80+) from stomach extract of WT and L22P mice with and without *H. pylori* infections. The experiment was done using a flow cytometer and data analysis was performed using FLowJo software; (**D**) qRT-PCR of macrophage mediated gene expression from levels from macrophage I (CXCL10 and NOS2) and Macrophage II (Arginase II) tissue extract derived from WT and L22P mice with or without H. pylori infection; (**E**) qRT-PCR of cytokine gene expression from WT and L22P mice with or without *H. pylori* infection. All statistical data analyses were processed using GraphPad prism. The statistical significance represented * *p* < 0.05; ** *p* < 0.01; *** *p* < 0.001; and **** *p* < 0.0001.

Oxidative DNA damage induced by *H. pylori* infection is a contributing factor for cancer initiation [22], which is associated with infiltrating immune cells including neutrophils, macrophages and direct effect of *H. pylori* interaction with host cells [23]. To gain insight into whether *H. pylori* infection induces Inducible nitric oxide synthase (iNOS) in macrophages, contributing to nitric oxide (NO) mediated DNA damage, we localized the macrophage that produces iNOS using immunocytochemistry. We detected an induction of iNOS production in macrophages in *H. pylori*-infected L22P versus WT gastric samples (Figure 3A). Strong immunoreactivity for iNOS was observed limited to the inflammatory cell infiltrate in the mucosa and deep mucosa in all infected samples. In contrast, uninfected mice samples were either negative or, when positive, only a very small number of iNOS-producing cells were detected in the lamina propria.

In addition, qRT-PCR data from stomach tissue extracts show that iNOS expression was increased 38-fold in L22P infected stomach tissue (Figure 3B, *p* < 0.001). To confirm our data, we isolated macrophages from WT and L22P stomach tissue and conducted flow cytometry (FACS) analysis after co-staining with antibodies that marks the surface of macrophage (F4/80) and iNOS. We found that the 65% of macrophages producing iNOS in *H. pylori* infected L22P mice versus infected WT mice (Figure 3C, *p* < 0.0001). This data suggests that *H. pylori* infection in combination with mutation in *POLB* enhances inducible iNOS in mouse stomachs. Furthermore, we examined which group of macrophage produces iNOS, so we measured the gene expression level of NOS2 and CXCL10 (macrophage I markers) and Arginase II (macrophage II marker). We found that *H. pylori* infection significantly increased gene expression of Arginase II in L22P mice (Figure 3D, *p* < 0.0001). In contrast, CXCL10 and NOS2 significantly decreased in L22P infected mice (Figure 3D; *p* < 0.0001). To determine whether infection related aberrant BER increases overall inflammatory response, we measured the expression levels of cytokines by qRT-PCR analysis in gastric tissues from WT and L22P mice. We found that the gene expression levels of IFNγ, TNF-α, IL-1β were significantly increased in *H. pylori* infected L22P mice versus infected WT mice (Figure 3E). This work suggests that the induction of iNOS in *POLB* mutant mice during *H. pylori* infection may play an important immunological role to induce stomach carcinogenesis.

### 2.4. H. pylori Infection Increases Tumor Incidence in POLB Mutant mice Independent of p53 Mutation

**Figure 4:**
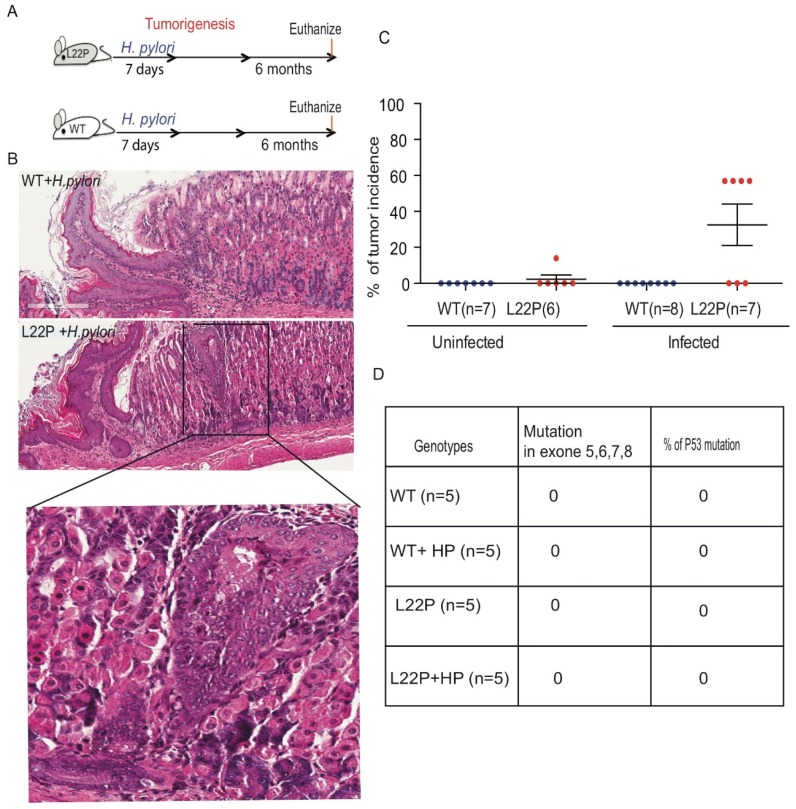
*H. pylori* infection increases tumor incidence and latency in *POLB* mutated mouse model. (**A**) Schematic representation of *H. pylori* mediated carcinogenesis study conducted for 3 months and 6 months after treatment with *H. pylori*; Five to eight weeks old WT and L22P mice were infected with *H. pylori* and evaluated for bacteria colonization after 7 days of infection and magnitude of inflammation was evaluated after 3 months of infection and all mice were terminated at 6 months of infection. (**B**) H and E staining of WT and L22P mice infected with *H. pylori*; image shows magnified (40x) pathological lesions of the inflammation on L22P gastric gland regions; (**C**) Estimated tumor incidence in WT and L22P with and without *H. pylori* infection after 6 months; (**D**) Percent of WT and L22P mice with p53 mutation in exon 5, 6, 7 and 8 with or without *H. pylori* infection.

To determine the impact of L22P on *H. pylori*-induced tumorigenesis, we infected L22P and WT mice and followed up for 6 months as shown in Figure 4A. L22P mice infected with *H. pylori* exhibited increased gastric inflammation compared to WT mice (bottom panel of Figure 4B). Histological analyses indicated that all infected WT mice had normal gastric glands (Figure 4B). Therefore, we extended our study using the *POLB* mutant mouse model to examine whether *H. pylori* infection could affect the incidence of gastric cancer in this model. None of the *H. pylori*-infected WT mice developed hyperplastic or dysplastic lesions (Figure 4C). In contrast, 57% of L22P mice developed gastric lesions following *H. pylori* infection (Figure 4C). The results clearly indicated that *H. pylori* infection in L22P mice significantly enhanced the incidence of gastric adenocarcinoma as compared to uninfected L22P mice within five months of exposure. Furthermore, to determine whether secondary mutation in tumor suppressor gene contributes to *H. pylori* induced gastric carcinogenesis, we sequenced exon 5, 6, 7 and 8 of p53 and found no mutation in both H. pylori infected L22P and WT infected mice, suggesting that p53 plays an independent role of POLB mutation induced carcinogenesis. These findings suggested that aberrant POLB is a critical factor for *H. pylori* to promote cancer development independent from p53 mutations.

## 3. Discussion

In this study, we found the role of aberrant BER in infection related genomic instability and carcinogenesis. Given the results presented above, our working hypothesis was that *H. pylori* infection results in RONS-induced DNA damage that is poorly repaired in the presence of mutant Pol β and that the unrepaired DNA lesions enhance tumorigenesis. This work used a Pol β mutant mouse model, which lacks dRP lyase activity and isogenic wild-type controls to evaluate tumor incidence and latency in carcinogenesis. Our data show that mutation in Pol β does not alter the rate of colonization of *H. pylori* in stomach tissue, suggesting that host-pathogen interaction and the development of bacterial colonies are likely not influenced by host BER function. Most importantly, our study shows that *H. pylori* infection in L22P mice has been associated with an increase in gastric epithelial cell proliferation, suggesting that an increased proliferation rate may promote the early stages of precancerous cellular transformation. Possibly, a disproportionate increase in proliferation relative to apoptosis in *H. pylori* infected Pol β mutant mice may increase the epithelial turnover and/or increase epithelial cell renewal. Interestingly, gastric epithelial cells and mucous layers produce Trefoil factor 1 and 2 (TFF1 and TFF2) and MUS5AC genes and were found overexpressed in *H. pylori* infected Pol β mutant mice rather than infected WT mice. Our findings are consistent with the data reported in other published studies where MUC5AC, which belongs to the gastric-type mucin, is strongly expressed in the surface epithelium along with TFF1 [24,25]. In addition, our data are consistent with previously published work that showed that TFF1 and TFF2 are frequently overexpressed in several human cancers [24,26]. This finding suggests that *H. pylori* interact with the mucin and non-mucin component of the gastric tissues in *POLB* mutant mice.

Several studies have reported that, DNA repair deficiency (miss match repair) induce mutation accumulation during *H. pylori* infection [27,28,29]. In addition, other studies have shown that BER is crucial for maintaining genomic stability to prevent carcinogenesis [30,31,32,33]. A tight coordination and efficiency of repair of RONS induced DNA lesions at different steps in BER is necessary to avoid genomic instability [34]. *H. pylori* infection in Pol β mutant mice significantly increases the number of 8-oxoG base damage versus infected WT mice, suggesting that accumulation of unrepaired BER intermediates may cause DSBs that subsequently lead to genomic instability and carcinogenesis. Our data is consistent with other previously published work that oxidative DNA damage contributes to gastric carcinogenesis [22]. This work is consistent with our co-culture experiment, which showed that normal human gastric epithelial cells versus mutant cells that are infected with *H. pylori* result in more BER intermediates and DSBs [35,36]. 

Chronic inflammation induces complex growth factors and cytokines by causing RONS production that leads to DNA damage. Our data show that *H. pylori* induces chronic inflammation may promote proliferation that likely act synergistically with RONS mediated DNA damage to enhance tumor development in Pol β mutant mice. Alternatively, Pol β mutant mice may accumulate unrepaired DNA damage that subsequently causes genomic instability to initiate carcinogenesis. Our data support both scenarios, since defects in Pol β function promote the accumulation of a significant amount of DNA damage (Figure 2). Furthermore, our data show that *H. pylori* infection in Pol β mutant mice enhances iNOS from macrophage cells similar to other pathogenic microorganisms in numerous diseases [37]. This observation supports the notion that NO production may increase and contribute to more RONS production in Pol β mouse stomach. Previously, we have seen that most bacteria activates iNOS in macrophages after invasion [38]. Our data are consistent with other studies that have shown that NO produced by iNOS in macrophages and other innate immune cells is an essential component of the host immune response against bacteria [39,40]. In addition, IL-1B and TNF-α levels have been shown to increase in *H. pylori*-infected Pol β mutant mouse gastric mucosa, which may stimulate gastric dysplasia and gastric cancer development. In contrast, in some studies, no relationship between *H. pylori* and TNF-α was observed [41]. However, our data are consistent with the work of Fan et al. [42], which showed that higher levels of TNF-α production by gastric mucosa cells in *H. pylori* infection may reflect the mucosal infiltration by T-lymphocytes and macrophages.

Studies in mice carrying *H. pylori* transgenic *cagA* gene induces gastric epithelial hyperplasia and some mice developed gastric polyps and adenocarcinomas of the stomach [20], further supporting a role for H. pylori in gastric carcinogenesis. Our work shows that *H. pylori* infection in BER deficient mice decrease tumor latency and increase tumor incidence suggesting that *POLB* is required to suppress *H. pylori* induced carcinogenesis. Interestingly, *H. pylori* infection in Pol β mutant mice decreases tumor latency independently of p53 mutations. Even though previous studies shows that mutations of *TP53* and *APC* genes were detected in intestinal metaplasia and gastric dysplasia [21,43], our data did not find mutation in TP53 (Figure 4), suggesting that *POLB* mutation may be the only factor to synergize tumor initiation independent of those tumor suppressor and oncogenes. The persistence of *H. pylori* infection in host cells causes a chronic inflammatory state with continued oxidative stress within the tissue that could lead to dysregulation of cell cycle proteins such as p53. Several studies have shown that *H. pylori*-induced dysregulation of p53 is a potential mechanism by which the microorganism increases the risk of gastric cancer in infected individuals. Our data contrast with the observations of Meiran et al. [44], who found that *H. pylori* infection induces tumor suppressor mutation and oncogene activation.

Based on our results, Pol β and other BER genes are critical candidates for genetic association studies of human gastric cancer risk. A gene polymorphism in DNA repair or promoter methylation of repair genes may affect the function and repair efficiency, which may further contribute to increased mutagenesis during gastric carcinogenesis [45,46]. Although there are few known polymorphisms that alter Pol β function, our findings suggest that Pol β dependent BER is required for repair of *H. pylori* induced oxidative DNA damage and suppresses inflammation-associated carcinogenesis. Our data clearly demonstrates that *H. pylori* associated RONS and inflammation-dependent DNA damage lead to tumor development in a repair-deficient genetic host. This may be particularly important for gene-environment interactions with states of chronic infection that induces chronic inflammation associated gastric cancer. Overall, this work provides molecular insight into Pol β mediated BER is critical to prevent *H. pylori* induced carcinogenesis.

## 4. Material and Methods

### 4.1. H. pylori Culture

The H. pylori J99 strain (ATCC 700824) is a human isolate that was used in the cell culture experiments due to its ability to strongly infect human cell lines. The *H. pylori* strain was inoculated onto Columbia blood agar plates, which consisted of 39 g of Columbia Agar Base (CM0331, Becton Dickinson, Franklin Lakes, NJ, USA) per liter and 7% hemolyzed horse blood (SR0048), supplemented with one vial of Dent (SR0147, Oxoid, Hampshire, UK). For growing visible colonies, plates were kept in a Gaspack (BD GasPak EZ products, Franklin Lakes, NJ, USA) at 37 °C for 3 to 10 days until colonies were observed. From the primary growth, single colonies were propagated in blood agar for an additional 48 h, harvested in phosphate-buffered saline (PBS) and inoculated into Hams F12 media supplemented with 5% horse serum and incubated in 5% CO_2_ incubator.

### 4.2. H. pylori Co-Culture Experiment

GES-1 cells were grown on four chamber slides and infected with *H. pylori* for 24 h (100:1 bacteria to host cell ratio, MOI = 100). Nuclear localization of γH2AX and cellular transformation assay was done as described previously [35]. 

### 4.3. Mice Experiment

For the *H. pylori* studies, animals were maintained in non-recyclable microisolator cages as previously described [47] and dirty bedding from WT and L22P mice were mixed to ensure all groups had similar commensal microbial flora. All mice were fed a standard diet ad libitum and housed in the Dell Pediatric Research Institute accredited facility. Euthanasia was by CO_2_ asphyxiation and all procedures were approved by the University of Texas at Austin Committee on Animal Care (Protocol number, AUP-2017-00068).

### 4.4. Establishing H. pylori Infection in WT and L22P Mice

*H. pylori* strain were cultured and prepared as described previously [35]. Mice at the age of 5 to 8 weeks were infected with a single dose, 2 × 10^7^ CFU, of *H. pylori*, that were orally administered to L22P and WT mice as described previously [47,48]. Control mice were dosed with broth media without *H. pylori*. The mice were euthanized at 12 and 24 weeks after inoculation and multiple gastric biopsies from infected and control stomachs were tested for the presence of bacteria after two weeks of infection, as described [49], For colonization assessment of bacteria in the stomach, we extracted the total genomic DNA and subjected to quantitative RT-PCR analysis of the 16S rRNA gene to calculate the copy number difference as a measure of for colonization as previously described [50]. 

### 4.5. Detection of H. pylori Using Immunofluorescence Assays

Tissue sections were deparaffinized by heating slides for 1h at 58 °C and incubation in xylene for 5 min. Sections were then rehydrated with 100% Ethanol, 95% Ethanol and water for 5 min in each solvent. Sections were incubated with Proteinase K (20 μg) for 30 min at room temperature to retrieve antigen and later rinsed with 1× PBS. After blocking the sections with 3% BSA for 30 min, the tissue sections were incubated with 1:100 dilution of primary anti-CagA antibody (Santa Cruz Biotechnology, Inc., sc-28368, Santa Cruz, CA, USA) in BSA at 4 °C overnight. Next day, sections were washed with 1× PBS three times and incubated with Secondary anti-FITC conjugated (1:400 in BSA) antibody for 2 h. Sections were washed with 1XPBS and stained with DAPI. Bacterial counts were analyzed using Image J and bacteria/mm^2^ mucosa was calculated by dividing the bacterial count by field size of the microscope (Field size = Field number/Objective magnification).

### 4.6. Mice Stomach Histology

Histological characterization will be performed based on the criteria of previously published work [44]. Stomach tissues were sectioned along the longitudinal axis into strips, washed in PBS and fixed in 10% neutral-buffered formalin solution (pH 7.4) overnight. Tissues were embedded in paraffin and serially sectioned at Dell Pediatric Research Institute Core Facilities. Immunohistology was conducted using the ImmunoCruz rabbit ABC Staining System (Santa Cruz, sc-2018). Primary antibodies for Ki-67 (Millipore, AB9260, Upstate NY, NY, USA), cleaved caspase-3 (R&D Systems, AF835, Minneapolis, MN, USA), 8-oxoG (Abcam, ab26874, Cambridge, MA, USA), γH2AX (Cell Signaling, 9718S, Danvers, MA, USA), p-ATM (Cell Signaling, 4526S) were used to stain the tissue section. Images of the sections were captured using Scanscope (Leica Biosystem, Vista, CA, USA). For quantification, 5 fields of each sample were selected and the number of positive cells were stained for those markers counted against the total number of cells in the selected fields. For F4/80, iNOS co-staining, sections were deparaffinized in xylene and rehydrated in a descending series of ethanol solutions. Antigen retrieval was performed by immersing sections in citric acid buffer (10 mM, pH 6.0) while microwaving for 20 min. Next, sections were blocked with 3% BSA for 30 min and immuno-staining was conducted using F4/80 (BD Pharmigen, 5121595, San Diego, CA, USA) and iNOS (Abcam, ab15323) overnight. The next day, sections were incubated for 90 min with Fluorescein isothiocyanate (FITC) conjugated anti-mouse antibody (Jackson immunoResearch Labs, 715-095-150, West Grove, PA, USA) and Tetramethylrhodamine (TRITC) conjugated anti-rabbit antibody (Jackson ImmunoResearch Labs, 711-025-152). Lastly, sections were washed with PBS and mounted with mounting media containing DAPI stain (Invitrogen, 1920964, Carlsbad, CA, USA). Images were captured using a Carl Ziess microscope with an MRc5 color camera.

### 4.7. Immunoblot

Stomach tissues were grinded in liquid nitrogen and lysed with Radioimmunoprecipitation assay (RIPA) lysis buffer supplemented with protease inhibitor (Sigma Aldrich, 25765800, St. Louis, MO, USA). After denaturing at 95 °C for 5 min, 30 μg of protein samples were separated by SDS-PAGE and transferred to nitrocellulose membranes (Bio-Rad, 1620112, Hercules, CA, USA). Next, membranes were blocked with 5% BSA for 1 hour and then incubated with primary antibody against γH2AX (Millipore, 07-164) and α-tubulin (Cell Signaling, 2125S) overnight. The next day, membranes were washed with PBST and incubated with anti-mouse (GE healthcare, NXA931, Chicago, IL, USA) or anti-rabbit (GE Healthcare, NA934V) secondary antibody for 120 mins before developing with ECL substrates (Bio-rad, 170506). The gel images were captured using a Chem-DocXRS (Bio-Rad). 

### 4.8. Preparation of Cell Suspension from Stomach Tissue

WT and L22P mice with and without *H. pylori* infection after 3 and 6 months were euthanized and stomach tissues were harvested and processed by combining about 50ng target tissue with 10 µL of 100 mg/mL Dispase/Collagenase (Sigma, 10269638001), 20 µL of 10 U/µL DNase (Sigma, 4716728001) in 1 mL PBS. This mixture was incubated at 37 °C for 30 min. Mixture was single cell separated by passing through a 35 µm cell strainer into a fresh 1.5 mL microtube. Wash 2× with 1 mL cold FACS Buffer (4% FBS in PBS) using centrifugation at 400× *g* for 5 min. at 4 °C. Resuspend cells in 100 µL FACS Buffer and keep on ice. For preparations of surface stains for F4/80 macrophage, we used F4/80 primary antibody (BD Pharmigen, 565409) was added to each sample resuspension and samples were incubated 1 h. on ice, then washed 2× at 400× *g* for 5 min. at 4 °C with 500 µL cold FACS Buffer. Next, samples were resuspended in cold FACS Buffer containing a 1:400 dilution of secondary antibody (JacksonImmResearch Labs., 712-095-153, West Grove, PA, USA). Samples were incubated 1 h. on ice, then washed 2× at 400× *g* for 5 min. at 4 °C with cold PBS. Samples were resuspended in 200 µL 0.5% formaldehyde in PBS for 15 min. at room temperature to fix the surface stains. For intracellular stain of iNOS, cells were permeabilized by permeabilization buffer (1% Triton-X in PBS). Samples were pelleted at 400× *g* for 5 min. Samples were resuspended in permeabilization buffer and then stained with iNOS primary antibody (Abcam, ab15323). Samples were incubated 30 min at RT, then washed 2× at 400× *g* for 5 min. Next, samples were resuspended in permeabilization buffer containing a 1:400 dilution of secondary antibody (JacksonImm., 711-165-152). Samples were incubated 30 min. at RT, then washed 2× at 400 ×g for 5 min. with 500 µL permeabilization buffer. Resuspended samples in 200 µL cold FACS buffer was used for analysis.

### 4.9. FACS Analysis

Samples were processed and flow cytometry analysis was done using a FACS Aria II and data was analyzed using Flow Jo software. Compensation was performed using unstained and single stained cell populations from each individual tissue or blood collection. Gating strategies were first to isolate singlets using FSC H × FSC A, isolate live immune cells using FSC A × SSC A and then to set positive gates using FMOs. Cell populations presented as percentages of live cell populations.

### 4.10. Quantitative Real-Time PCR

Tissue samples were homogenized in Eppendorf tubes using pestle on ice. RNA was extracted using 1mL Trizol (Thermo Fisher, 15596026, Carlsbad, CA, USA) reagent. cDNA was synthesized from 2 µg of RNA using High-Capacity cDNA Reverse Transcription Kit (Applied Biosystem, 4368814, Carlsbad, CA, USA). For the amplification of the target genes, 100 ng of cDNA was used in the final reaction mixture of 20 µL, with 10 µL of iTaq^TM^ Universal 2× SYBR^®^ green supermix (172-5121, Bio-Rad, Hercules, CA, USA), 500 nM of Forward and Reverse Primers. The samples were run in a ViiA7 Real-Time PCR System (Applied Biosystems, Carlsbad, CA, USA) in 384 well plates. The relative expression of gene was calculated by 2^(−∆∆CT)^ method and Glyceraldehyde 3-phosphate dehydrogenase (GAPDH) was used as an internal control. All primers used for this study listed on Supplementary Materials Tables S1 and S2.

### 4.11. Statistical Analyses

GraphPad Prism software (GraphPad Prism, version 8, San Diego, CA, USA) was used for statistical analyses. For *H. pylori* studies, group means of serum antibody titers and log-transformed bacterial colonization data were compared using a student’s *t* test. All *p* values listed are 2 sided except in our comparison of tumor incidence. Data are mean ± SEM. A *p* value <0.05 was considered significant.

## 5. Conclusions

Here, we show that aberrant DNA polymerase beta significantly promote genomic instability in *H. pylori* infected cells. We also showed that inflammatory response significantly increases in aberrant POLB mouse model. These data suggested that aberration base excision repair might contribute to decrease tumor latency and increase the tumor incidence in *H. pylori* infected groups. 

## Supplementary Materials

The following are available online at https://www.mdpi.com/2072-6694/11/6/843/s1, Figure S1: Apoptosis moderately increase in aberrant BER mice, Table S1: List of primers for gene expression, Table S2: List of primers for mutation analysis.
