# Supplementary material for: Aberrant DNA Polymerase Beta Enhances H. pylori Infection Induced Genomic Instability and Gastric Carcinogenesis in Mice
Source: Cancers (Basel). 2019 Jun 18;11(6):843. doi: 10.3390/cancers11060843 (PMC6627457; doi:10.3390/cancers11060843)
Supplement: Supplementary file 1 [file cancers-11-00843-s001.pdf]

cancers

Article

Aberrant DNA Polymerase Beta Enhances *H. pylori* Infection Induced Genomic Instability and Gastric Carcinogenesis in Mice

Shengyuan Zhao, Megha Thakur, Alex Klattenhoff, and Dawit Kidane

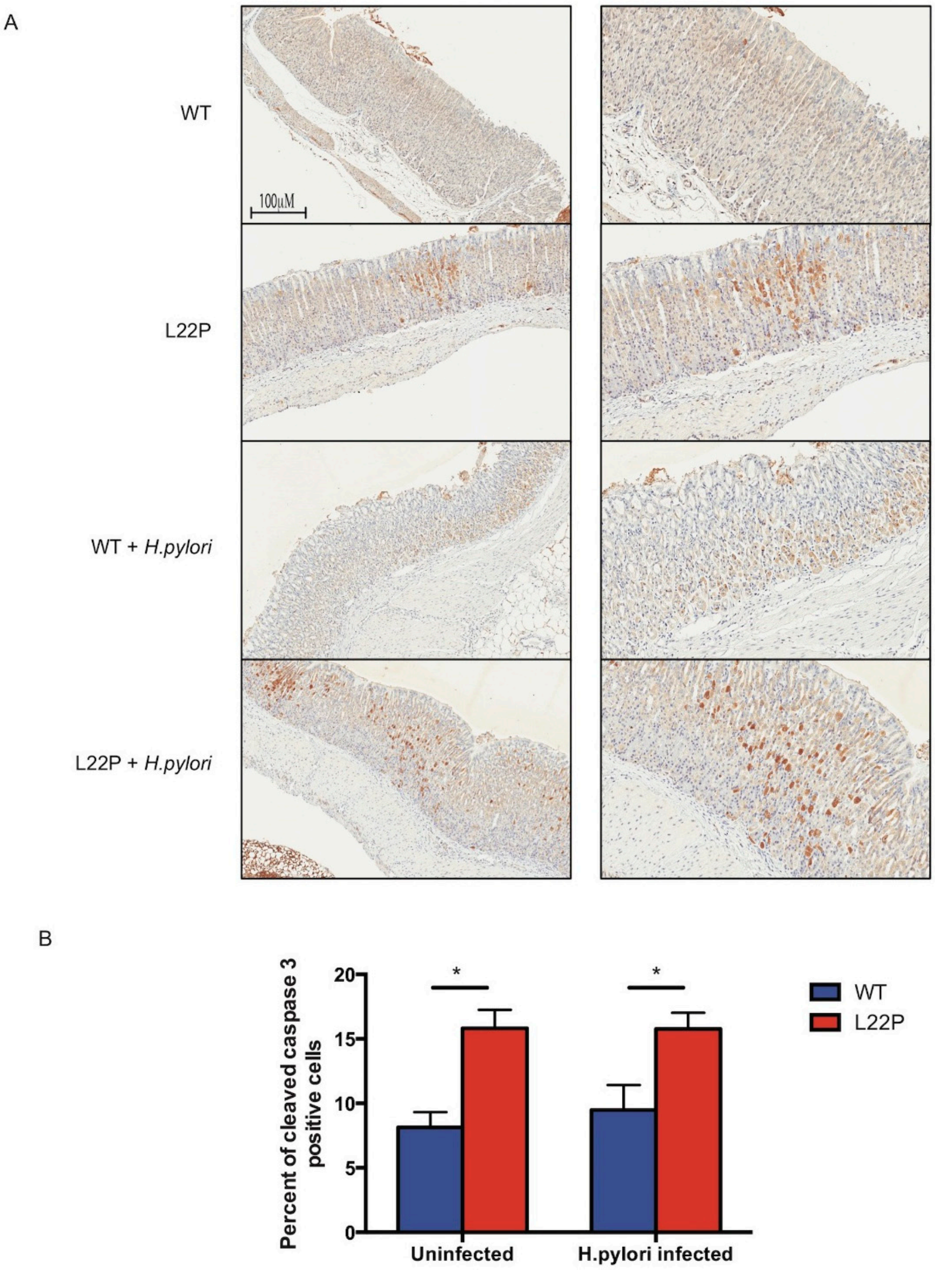

Figure S1. Apoptosis moderately increase in aberrant BER mice. (**A**) Representative image of stomach tissues stained with cleaved caspase-3 antibody; (**B**) Estimated percent of positive stained cells in WT and L22P with and without *H. pylori* infection. All statistical analysis was performed using GarphPad prism software (version 8, GraphPad Prism Software Inc. San Diego, CA, USA).

Table S1. List of primers for measuring gene expression using quantifying RT-PCR.

Gene		Gene Sequence (5′-3′)
GAPDH	Forward	TGCACCACCAACTGCTTAG
	Reverse	GGATGCAGGGATGATGTTC
IFNγ	Forward	CCAGGACCCATATGTAAAAGAAGC
	Reverse	TCATGTCTTCCTTGATGGTCTCC
IL-6	Forward	ACAAAGCCAGAGTCCTTCAGAGA
	Reverse	CTGTTAGGAGAGCATTGGAAATTG
IFNβ	Forward	AGAAAGGACGAACATTCGGAAA
	Reverse	TCCGTCATCTCCATAGGGATCTT
iNOS	Forward	CGAAACGCTTCACTTCCAA
	Reverse	TGAGCCTATATTGCTGTGGCT
TNF-α	Forward	GTAGCCCACGTCGTAGCAAA
	Reverse	ACAAGGTACAACCCATCGGC
IL-1β	Forward	CAGGGCTGTGCTGATTGAG
	Reverse	CGGACATACTTGAGGCTGTTC
CXCL10	Forward	GGATGGCTGTCCTAGCTCTG
	Reverse	TGAGCTAGGGAGGACAAGGA
NOS2	Forward	TTACGTCCATCGTGGACAGC
	Reverse	TGGGCTGGGTGTTAGTCTTA
Arginase II	Forward	ATATGGTCCAGCTGCCATTCGAGA
	Reverse	TAACCACTTCAGCCAGTTCCTGGT
16s rRNA	Forward	AGAGTTTGATCCTGGCTCAG
	Reverse	GGTTACCTTGTTACGACTT

Table S2. List of primers for sequencing of genes.

Exon		Gene Sequence (5′-3′)	Amplicon size
p53-exon 5	Forward	CCCCACCTTGACACCT	300 bp
	Reverse	CCGGGATATGGGAGGC	
	Sequence	TCTTCCAGTACTCT	
p53-exon 6	Forward	TCTCCCGGCTTCTGACTTA	300 bp
	Reverse	CTCCCAGAGACTGCTGTTA	
	Sequence	TTCTTGCTCTTAGGCCT	
p53-exon 7	Forward	TCTGAGTATACCACCATCCA	250 bp
	Reverse	ACAGATAAGAGACGTCCCTA	
	Sequence	CTACAAGTACATGTGTAAT	
p53-exon 8	Forward	TCCTTTTCTTGTCCCGGA	280 bp
	Reverse	GGTGAAATACTCTCCATCA	
	Sequence	TAGTGGGAACCTTCTGG	
ctnnb1-exon 2	Forward	GCTGACCTGATGGAGTTGGA	227 bp
	Reverse	GCTACTTGCTCTTGCGTGAA	
	Sequence	GCTCTTGCGTGAAGGACT	
Kras–exon 1, 2	Forward	GCCTGCTGAAAATGACTGAGTA	1600 bp
	Reverse	CAAGTCATGTAACTGCTGTAAC	
	Sequence – exon 1	CTTGCACCTATGGTTCCCTAA	
	Sequence – exon 2	CAGACTGTGTTTCTCCCTTCTC	

© 2019 by the authors. Submitted for possible open access publication under the terms and conditions of the Creative Commons Attribution (CC BY) license (http://creativecommons.org/licenses/by/4.0/).
